# Pregnant mothers are more anemic than lactating mothers, a comparative cross-sectional study, Bahir Dar, Ethiopia

**DOI:** 10.1186/s12878-018-0096-1

**Published:** 2018-01-16

**Authors:** Berhanu Elfu Feleke, Teferi Elfu Feleke

**Affiliations:** 1 Department of Epidemiology and Biostatistics, University of Bahir Dar, Bahir Dar, Ethiopia; 2 Departement of pediatrics, saint paulose hospital, Addis Ababa, Ethiopia

**Keywords:** Anemia, Determinants, Lactation, Pregnancy, Prevalence

## Abstract

**Background:**

Information on the hemoglobin status of pregnant and lactating mothers was scarce. The objectives of this study were to determine the burden and determinants of anemia in the pregnant and lactating mother.

**Methods:**

A comparative cross-sectional study was conducted. Descriptive statistics were used to identify the prevalence of anemia. Binary logistic regression and multiple linear regressions were used to identify the predictors of anemia.

**Results:**

The prevalence of anemia in lactating and pregnant women was 43.00% (95% CI {confidence interval}, 41% - 45%) and 84% of anemia was microcytic and hypocromic anemia. Anemia in lactating and pregnant women was positively associated with malaria infection [AOR{adjusted odds ratio} 3.61 (95% CI: 2.63–4.95)], abortion [AOR 6.63 (95% CI: 3.23–13.6)], hookworm infection [AOR 3.37 (95% CI: 2.33–4.88)], tea consumption [AOR 3.63 (95% CI: 2.56–5.14)], pregnancy [AOR 2.24 (95% CI: 1.57–3.12)], and Mid-upper arm circumference [***B*** 0.36 (95% *CI:* 0.33, −0.4)]. Anemia in pregnant and lactating mother was negatively associated with urban residence [AOR 0.68, (95% CI: 0.5–0.94)], iron supplementation during pregnancy [AOR 0.03 (95% CI, 0.02–0.04)], parity [***B*** -0.18 (95% CI: -0.23, −0.14)], age [B -0.03 (95% *CI:* -0.04, −0.03)].

**Conclusion:**

The burden of anemia was higher in pregnant women than lactating women.

## Background

Anemia is a condition in which the hemoglobin concentration of a woman is less than 11 g/dl (gram per deciliter). World health organization report indicated that 20–50% of the world population was affected by iron deficiency anemia [1, 2]. Anemia was one of the great public health burdens for pregnant women affecting 56 million pregnant women globally [3, 4]. In the developed countries, 18% of pregnant women were anemic but in developing countries, 35–75% of pregnant women were anemic [5, 6].

Anemia during pregnancy has many adverse outcomes for the mother and her child. Globally, anemia resulted in the death of 115,000 mothers and 591,000 perinatal mortality annually [7–12]. Anemia during pregnancy predisposes mother to prolonged labor, abnormal delivery, increases the risk of hemorrhage [13–15]. Also, anemia increases the risk of infection to pregnant women due to its effect on the immune system [11, 16–20]. Newborns receive a number of complications as a result of maternal anemia. Among others, maternal anemia increases the risk of perinatal mortality and morbidity, preterm deliveries, low birth weight baby, intrauterine growth retardation (IUGR) [21–31].

The burden of anemia varies from country to country; in Germany, 51% of pregnant women were anemic, in Trinidad and Tobago 15%, in Nepal 72.6% and 58% in India [32–35]. In Africa, more than 60% of pregnant women were anemic: in Ghana 44% of pregnant women, in Benin 24.3% of pregnant women, in Kenya 69.1% of pregnant women and in eastern Sudan 80.3% of pregnant women were anemic [36–42]. Ethiopia is among country highly affected by anemia: the prevalence of anemia among pregnant women ranges from 15%-63%, in lactating mothers the prevalence of anemia was 22.3% [4, 43–49].

Finding from different scholars globally revealed that anemia in pregnancy was associated with gestational age, iron supplementation during pregnancy, wealth quintile, gravidity, mid-upper arm circumference (MUAC), age, residence, history of malaria infection, hookworm infection, parity, history of abortion, chronic inflammatory disorders, use of insecticide-treated bed net, tea consumption and use of animal product [33, 34, 39, 41, 42, 48–55].

Even if anemia resulted in these entire adverse outcomes for pregnant mother and their children, information on hemoglobin status of pregnant and lactating mothers are scarce. Due to lack of information decision makers are not capitalizing on the problem of anemia in pregnancy and lactating mother. This study is designed to attempt to fill these gaps. The objectives of this study were to compare the prevalence of anemia among pregnant and lactating women. Furthermore, the study attempted to identify the determining factors of anemia in pregnant and lactating mother and these aims were achieved successfully.

## Methods

Health facility-based comparative cross-sectional study was conducted. The study was conducted in the city of Bahir dar, the capital of the Amhara regional state, located at the geographical coordinates of 11° 38′ north latitude and 37° 15′ east longitude, which is located approximately 560 km (km) northwest of Addis Ababa. The target populations were all pregnant and lactating mother in the city of Bahir dar and the study population were those presenting themselves for medical help. Pregnant or lactating mother unable to communicate were excluded from the study. The sample size was calculated using Epi-info software version 7 using the assumption of 95% confidence interval, pregnant to lactating mother ratio of 2:1, the proportion of anemia among lactating mother 22.3% [46], a power of 90%, an odds ratio of 1.5 and a non-response rate of 10%. Finally, the estimated sample size was 567 pregnant women and 1133 lactating women. Study participants were selected from health facilities of Bahir dar city. Stratified sampling technique was used to select study participants from each health facility. The data were collected from November 2014–May 2015. The data collection procedures contained two parts, exit interview and collecting blood and stool samples. For the interview part, first, the questionnaire was prepared in English then translated to Amharic (local language) then back to English to keep its consistency. The interview was conducted by 10 nurses professional and supervised by 3 health officers. The blood and stool samples were collected by 5 first degree holder laboratory technologists and supervised by two second degree holder laboratory technologists. From each woman, one gram stool sample was collected in 10 ml (ML) SAF (sodium acetate- acetic acid-formalin solution). Concentration technique was used. The stool sample was well mixed and filtered using a funnel with gauze then centrifuged for one minute at 2000 RPM (revolution per minute) and the supernatant was discarded. 7 ML normal saline was added, mixed with a wooden stick, 3 ML ether was added and mixed well then centrifuged for 5 min at 2000 RPM. Finally, the supernatant was discarded and the whole sediment was examined for parasites [56]. 1 ML blood sample was collected from each woman following standard operational procedures to measure their hemoglobin level and red blood cell indices using Mindray hematology analyzer. To maintain the quality of the data; pretest was conducted in 50 parents, training was given to data collectors and supervisors and the whole data collection process was closely supervised by the investigator and supervisors. The collected data were checked for completeness. The data were entered into the computer using Epi-info software and analyzed using statistical package for social sciences (SPSS) software. Descriptive statistics were used to estimate the prevalence of anemia among pregnant and lactating women. Binary logistic regression and multiple linear regressions were used to identify the determinants of anemia.

Ethical clearance was granted from Amhara National Regional State Health Bureau ethical committee. Legal permission was obtained from each health center. Written informed consent was obtained from each study participant. The confidentiality of the data was kept at all steps. Women with intestinal parasites or low hemoglobin concentration (<11 g/dl) were referred to the nearby health center for further management.

## Results

A total of 1651 women was included giving a response rate of 97.12%. The mean age of the respondents was 22.65 years (SD [standard deviation] 5.12 years). Orthodox Christian constituted 96.7% (1596) of study participants, 90.1% of study participants were Amhara by ethnicity, 52.7% of women were from urban areas, and 49.3% of study participants were house wife by their occupation (Table 1).

**Table 1:** Socio-demographic characteristics of study participants (*n* = 1651)

*SN*	*Population profile*		*Frequency*	*Percentage*
1.	Age	16–25	1279	77.5
		26–35	316	19.1
		36–45	56	3.4
2.	Residence	Urban	865	52.4
		Rural	786	47.6
3.	Religion	Orthodox	1596	96.7
		Muslim	45	2.7
		Protestant	10	0.6
4.	Ethnicity	Amhara	1488	90.1
		Agaw	110	6.7
		Oromo	13	0.8
		Tigray	23	14
		Others	17	1
5.	Educational status	Illiterate	40	2.4
		Elementary	408	24.7
		Secondary	318	19.3
		Certificate	399	24.2
		Diploma	242	14.7
		First degree	227	13.7
		Second degree	17	1
6.	Occupation	Housewife	814	49.3
		Government employee	306	16.5
		Merchants	161	9.8
		Farmers	156	9.4
		NGO employee	157	9.5
		Others	57	3.5

### Anemia in pregnant women

A total of 550 pregnant women was included giving a response rate of 97%. The mean age of pregnant women was 26.88 years (SD = 5.82 years). After adjusting for women’s residence, history of abortion, history of malaria, occupation, hookworm infection, tea consumption and iron supplementation during pregnancy: the risk of anemia increases in rural women, history of abortion or malaria, hookworm infection, and tea consumptions. The risk of anemia was lower in women with government employer, in women that were supplied by iron during pregnancy (Table 2).

**Table 2:** Binary logistic regression output of determinants of anemia during pregnancy (*n* = 550)

*Variables*		*Anemia*		*COR[95% CI]*	*AOR[95% CI]*	*p-value*
		*Yes*	*No*			
*Residence*	Urban	32	261	0.08 [0.05–0.13]	0.14 [0.08–0.25]	<0.01
	Rural	155	102			
*History of malaria*	Yes	83	63	3.8 [2.51–5.76]	2.84[1.65–4.92]	<0.01
	No	104	300			
*History of abortion*	Yes	27	9	6.64 [2.9–15.59]	4.44 [1.58–12.05]	0.01
	No	160	354			
*Hookworm infection*	Present	78	20	12.27 [6.97–21.78]	5.97 [3.03–11.76]	<0.01
	Absent	109	343			
*Iron supplementation during pregnancy*	Supplied	141	343	0.18 [0.1–0.32]	0.12 [0.06–0.25]	<0.01
	Not supplied	46	20			
*Tea consumption*	Yes	110	68	6.2 [4.11–9.37]	2.88 [1.54–5.38]	<0.01
	No	77	295			
*Occupation*	Government employ	22	96	0.37 [0.22–0.63]	0.35 [0.18–0.69]	<0.01
	Others	165	267			

*COR* crude odds ratio, *AOR* adjusted odds ratio

On linear regression anemia in pregnancy was associated with age, gravidity, mid-upper arm circumferences (MUAC) and gestational age (Table 3).

**Table 3:** linear regression output for determinants of hemoglobin concentration in pregnancy (dependent variable = hemoglobin concentration in g/dl)

*Variables*	*B coefficient [95% CI]*	*t*	*p-value*
MUAC	0.37 [0.32, 0.41]	15.55	<0.01
Gravidity	−0.35[−0.42,-0.29]	−10.91	<0.01
Age of pregnant mother	0.03 [0.02,0.03]	6.69	<0.01
Gestational age	−0.02[−0.03,-0.01]	−3.73	<0.01

*B* beta coefficient

The degree of anemia defers with the gestational age of pregnant mothers, per one week increase in the age of gestation her hemoglobin concentration will decrease by 0.02 g/dl. That means the higher the gestational age the risk of becoming anemic will also become high.

### Anemia in lactating women

A total of 1101 lactating women were included with a response rate of 97.18%. The mean age of the lactating women was 20.54 years (SD 3.12 years). On binary logistic regression after adjusting for residence, history of malaria, history of abortion, hookworm infection, iron supplementation, tea consumption: anemia in lactating women was associated with a residence, history of malaria, history of abortion, iron supplementation and tea consumption (Table 4).

**Table 4:** Logistic regression output of determinants of anemia in lactating women (*n* = 1101)

*Variables*		*Anemia*		*COR[95% CI]*	*AOR[95% CI]*	*p-value*
		*Yes*	*No*			
*Residence*	Urban	228	344	0.53 [0.41–0.67]	4.03 [2.3–7.03]	<0.01
	Rural	295	234			
*History of malaria*	Yes	274	122	4.11 [3.13–5.40]	4.73 [3.02–7.41]	<0.01
	No	249	456			
*History of abortion*	Yes	50	9	4.62 [2.16–10.18]	7.44 [2.3–24.09]	<0.01
	No	569	473			
*Iron supplementation during pregnancy*	Supplied	45	494	0.02 [0.01–0.02]	0.007 [0.004–0.013]	<0.01
	Not supplied	478	84			
*Tea consumption*	Yes	184	108	2.37 [1.78–3.15]	2.32 [1.48–3.64]	<0.01
	No	338	470			

On linear regression hemoglobin concentration in pregnancy was associated with MUAC, parity, age, and frequency of breastfeeding per 24 h (Table 5).

**Table 5:** Linear regression output for determinants of hemoglobin concentration in lactating mothers (dependent variable = hemoglobin concentration in g/dl) (n = 1101)

*Variables*	*B coefficient [95% CI]*	*t*	*P-value*
*MUAC*	0.25 [0.21, 0.29]	11.08	<0.01
*Parity*	−0.14 [−0.21, −0.06]	−3.64	<0.01
*Age of lactating mother*	−0.03[−0.05, −0.01]	−3.43	<0.01
*Frequency of breast feeding*	−0.28 [−0.33, −0.24]	−12.96	<0.01

### Anemia in pregnant and lactating women

The prevalence of anemia in lactating and pregnant women was 43% (95% CI, 41%-45%), 84% of anemia was microcytic hypocromic, 4.54% of anemia was macrocytic hypercromic, and 5.82% of anemia was normocytic normocromic (Table 6).

**Table 6:** The red blood cell indices of anemic women (*n* = 705)

Mean corpuscular volume (MCV)	Mean corpuscular hemoglobin concentration (MCHC)			Total
	Normocromic	Hypocromic	Hypercromic	
Normocytic	41	2	3	46
Microcytic	13	592	12	617
Macrocytic	6	4	32	42
Total	60	598	47	705

After adjusting for residence, pregnancy, history of malaria, history of abortion, hookworm infection, iron supplementation during pregnancy, tea consumption, occupation and educational status; anemia in pregnant or lactating mother were associated with a residence, pregnancy, history of malaria, history of abortion, hookworm infection, iron supplementation during pregnancy and tea consumption (Table 7).

**Table 7:** Logistic regression output of determinants of anemia in pregnant and lactating women (n = 1651)

*Variables*		*Anemia*		*COR[95%CI]*	*AOR[95%CI]*	*P-value*
		*Yes*	*No*			
*Residence*	Urban	260	605	0.32 [0.26–0.40]	0.68 [0.5–0.94]	0.02
	Rural	450	336			
*History of malaria*	Yes	357	185	4.13 [3.3–5.17]	3.61 [2.63–4.95]	<0.01
	No	353	756			
*History of abortion*	Yes	77	18	6.24 [3.61–10.91]	6.63 [3.23–13.6]	<0.01
	No	633	923			
*Hookworm infection*	Present	228	119	3.27 [2.53–4.22]	3.37 (2.33–4.88)	<0.01
	Absent	482	822			
*Iron supplementation during pregnancy*	Supplied	186	837	0.04 [0.03–0.06]	0.03 [0.02–0.04]	<0.01
	Not supplied	524	104			
*Tea consumption*	Yes	295	176	3.09 [2.46–3.88]	3.63 [2.56–5.14]	<0.01
	No	415	765			
*Pregnant/lactating*	Pregnant women	187	363	0.57 [0.46–0.71]	2.24 [1.57–3.12]	<0.01
	Lactating women	523	578			

On linear regression determinants of anemia in pregnant or lactating women were associated with mid-upper arm circumferences, age, and parity of pregnant or lactating women (Table 8).

**Table 8:** Linear regression output for determinants of hemoglobin in lactating or pregnant mothers (dependent variable = hemoglobin level in g/dl) (n = 1651)

*Variables*	*B coefficient [95% CI]*	*t*	*p-value*
MUAC	0.36 [0.33, −0.4]	21.66	<0.01
Parity	−0.18 [−0.23, −0.14]	−7.69	<0.01
Age of lactating/pregnant mother	−0.03[−0.04, −0.03]	−7.59	<0.01

## Discussion

The prevalence of anemia in lactating and pregnant women was 43% (95% CI, 41%-45%) and 84% of anemia was iron deficiency anemia followed by normocytic normocromic anemia This finding is lower when compared to findings from eastern Ethiopia [48] Germany [32], Nepal [33], India [35], eastern Sudan [40], Kenya [41]; agrees with findings from Ghana [39]and higher than finding from northern Ethiopia [49] southern Ethiopia [45] Trinidad and Tobago [34], Benin [42]. These might be due to the reason that different distribution of determinants of anemia across different social, cultural or geographical areas.

The risk of anemia in rural Lactating or pregnant women was 32% higher as compared to urban lactating or pregnant women [AOR 0.68, (95% CI: 0.5–0.94)]. This finding agrees with finding from northern Ethiopia [49]. This is due to the reason that women in the rural areas are in low socio-economic status so that they have no access to use iron rich foods [48].

Malaria infected women had 3.61 folds higher risk of anemia as compared to women with no history of malaria infection [AOR 3.61(95% CI: 2.63–4.95)]. This finding agrees with finding from north Ethiopia [49], Nepal [33], Ghana [52]. This is due to the fact that Plasmodium species ingests the red blood cells of the host and finally decreases the number of red blood cells.

Abortion increases the risk of anemia by 6.63 folds higher [AOR 6.63 (95% CI: 3.23–13.6)]. This finding agrees with finding from Trinidad and Tobago [34]. This is due to the reason that abortion increases the risk of hemorrhage.

Hookworm infection increases the risk of anemia by 3.37 folds higher [AOR 3.37 (95% CI: 2.33–4.88)]. This finding agrees with findings from northern Ethiopia [49], Nepal [33]. This is due to the fact that hookworm causing parasites significantly depletes the red blood cell of the host.

Iron supplementation during pregnancy decreases the risk of anemia by 97% [AOR 0.03 (95% CI: 0.02–0.04)]. The main reason for not receiving iron during pregnancy was unavailability of the drug. This finding agrees with finding from eastern Ethiopia [48]. This is due to the reason that iron act as a predominant role in the production of red blood cells.

Tea consumption increases the risk of anemia 3.63 folds higher [AOR 3.63 (95% CI: 2.56–5.14)]. This finding agrees with finding from Ethiopia [55]. This is because the fact that tea contains chemicals that inhibit the absorption of iron [57].

Pregnant mother had 2.24 folds higher risk of anemia than lactating mother [AOR 2.24 (95% CI: 1.57–3.12)]. This is due to the reason that after the delivery the mother has access to foods, especially animal products so that they can get more foods than when she was pregnant. In addition, mother can be treated for hookworm after delivery; during pregnancy, hookworm was not treated because the drug has a teratogenic effect.

MUAC had a positive relationship with hemoglobin concentration. Mid-upper arm circumference (MUAC) increase the hemoglobin concentration of women will also increase [***B*** 0.36 (95% *CI:* 0.33, −0.4)]. This finding agrees with finding from eastern Ethiopia [48] signaling that MUAC can be used to evaluate the nutritional level of pregnant or lactating women.

As the parity of women increases their hemoglobin concentration decreases [***B*** -0.18 (95% CI: -0.23, −0.14)].

This finding agrees with finding from the republic of Seychelles [53], Trinidad and Tobago [34], Benin [42]. This is due to the reason that as the number of pregnancy increases the risk of ante-partum hemorrhage and postpartum hemorrhage for the women became high.

The age of the women and her hemoglobin concentration had negative relationships. As the age increases the risk of becoming anemic would be high [B -0.03 (95% *CI:*-0.04, −0.03)]. This finding agrees with finding from Benin [42], northern Ethiopia [49]. This is due to the reason that as the age of the mother increases her parity wills also increases.

The main limitation of this study might be recall bias, but the interview was conducted using a structured questionnaire and the interviewers were trained health professionals they can probe and make the respondents remember the issues.

## Conclusion

Both pregnant and lactating mothers were affected by anemia and the burden of anemia is higher in the pregnant mother than the lactating mother and iron deficiency anemia is the most common type of anemia. Anemia in pregnancy and lactation was determined by a history of malaria, history of abortion, hookworm infection, tea consumption, MUAC, residence, iron supplementation during pregnancy, parity, and age.

## Recommendation

Iron supplementation should be given both to pregnant and lactating mothers. Iron supplementation should be included as part of malaria treatment in women with malaria. Women are advised to avoid tea during their pregnancy and lactation period. Scholars should consider MUAC as an alternative tool to detect nutritional defects in pregnancy.

## Abbreviation

AOR: Adjusted Odds Ratio
B: Beta Coefficients
CI: Confidence Interval
COR: Crude Odds Ratio
G/DL: Gram per Deciliter
IUGR: Intra Uterine Growth Retardation
KM: Kilometer
MCHC: Mean Corpuscular Hemoglobin Concentration
MCV: Mean Corpuscular Volume
ML: Milliliter
MUAC: Mid Upper Arm Circumference
RPM: Revolution per Minute
SAF: Sodium Acetate- Acetic Acid-Formalin Solution
SD: Standard Deviation
SPSS: Statistical Package for Social Science
